# Immunomodulating Activity of *Aronia melanocarpa* Polyphenols

**DOI:** 10.3390/ijms150711626

**Published:** 2014-06-30

**Authors:** Giang T. T. Ho, Marie Bräunlich, Ingvild Austarheim, Helle Wangensteen, Karl E. Malterud, Rune Slimestad, Hilde Barsett

**Affiliations:** 1 School of Pharmacy, Department of Pharmaceutical Chemistry, University of Oslo, P.O. Box 1068, Blindern, N-0316 Oslo, Norway; E-Mails: p.m.braunlich@farmasi.uio.no (M.B.); ingvild.austarheim@farmasi.uio.no (I.A.); helle.wangensteen@farmasi.uio.no (H.W.); k.e.malterud@farmasi.uio.no (K.E.M.); hilde.barsett@farmasi.uio.no (H.B.); 2 Norwegian Institute for Agricultural and Environmental Research—Bioforsk Vest Særheim, Postvegen 213, N-4353 Klepp station, Norway; E-Mail: rune.slimestad@bioforsk.no

**Keywords:** *Aronia melanocarpa*, anthocyanins, procyanidins, complement system, macrophages, nitric oxide, immunomodulation

## Abstract

The immunomodulating effects of isolated proanthocyanidin-rich fractions, procyanidins C1, B5 and B2 and anthocyanins of *Aronia melanocarpa* were investigated. In this work, the complement-modulating activities, the inhibitory activities on nitric oxide (NO) production in LPS-induced RAW 264.7 macrophages and effects on cell viability of these polyphenols were studied. Several of the proanthocyanidin-rich fractions, the procyanidins C1, B5 and B2 and the cyanidin aglycone possessed strong complement-fixing activities. Cyanidin 3-glucoside possessed stronger activity than the other anthocyanins. Procyanidins C1, B5 and B2 and proanthocyanidin-rich fractions having an average degree of polymerization (PD) of 7 and 34 showed inhibitory activities on NO production in LPS-stimulated RAW 264.7 mouse macrophages. All, except for the fraction containing proanthocyanidins with PD 34, showed inhibitory effects without affecting cell viability. This study suggests that polyphenolic compounds of *A. melanocarpa* may have beneficial effects as immunomodulators and anti-inflammatory agents.

## 1. Introduction

With recent advances in the understanding of how cells communicate with each other to signal effector functions, it has become possible to think of strategies to manipulate these signaling pathways in order to influence host responses. Compounds that are capable of interacting with the immune system to upregulate or downregulate specific aspects of the host response can be classified as immunomodulators or biologic response modifiers [1]. Several classes of compounds, such as polyphenols, proteins, polysaccharides, peptides, lipopolysaccharides, glycoproteins, and lipid derivatives, have been shown to contain molecules with potent effects on the host immune system by either enhancing or suppressing immune responses. Immunomodulatory activities have also been shown for a number of plant extracts and natural products used in traditional medicine, providing a rational explanation for their medicinal application [2].

Complement is an important part of the innate immune system, but has also been shown to be closely linked to the adaptive immune system [3]. The complement system comprises a group of serum proteins and cell membrane receptors that function primarily to fight infections. It participates in processes such as lysis of foreign cells, inflammation and phagocytosis [4].

Macrophages and their circulating monocyte form are potent defenders of physiological integrity by their mediation of crucial physiological and protective functions such as innate immunity and inflammatory reactions, foreign antigen presentation, and scavenging of dead cells [5]. Macrophages have the unique ability to metabolize one amino acid, arginine, to nitric oxide (NO) [6]. NO production endows macrophages with cytotoxic activity against bacteria, viruses, fungi, protozoa, and tumor cells [5]. In addition, NO plays an important role in the regulation of other physiological functions including neurotransmission, vasodilatation, and neurotoxicity [7]. However, excessive production of NO could be related to the development of septic shock, neuropathological diseases, as well as rheumatoid arthritis and other autoimmune disorders [5]. Therefore, inhibition of NO production may have potential therapeutic value when related to inflammatory diseases. 

*Aronia melanocarpa*, also called black chokeberry, is a member of the Rosaceae family. Shrubs of the aronia genus are native North American plants that have been traditionally used in Native American medicine, for example in the treatment of colds [8,9]. The aronia plant has become popular in recent years due to its berries having a high content of polyphenols with antioxidant activity. Aronia products are well received as nutritional supplements, and the berries are also used as an ingredient for juices, wines, jams, and as a source of natural food colorants [9].

While some studies on the effect of anthocyanins, proanthocyanidins and extracts rich in these compounds have been published [10,11,12,13], the knowledge on aronia extract and pure substances from the plant in regard to effects on the human complement system and NO production in LPS-stimulated macrophages is scant [14,15]. Thus, the present study was undertaken to determine the complement-modulating activities and the inhibitory activities on NO production in LPS-induced RAW 264.7 macrophage cells of aronia polyphenols. In order to get a broader view of the activity of the fractions and substances, two different assays were chosen for this investigation.

## 2. Results and Discussion

### 2.1. Fractionation and Chemical Characterization

**Figure 1:**
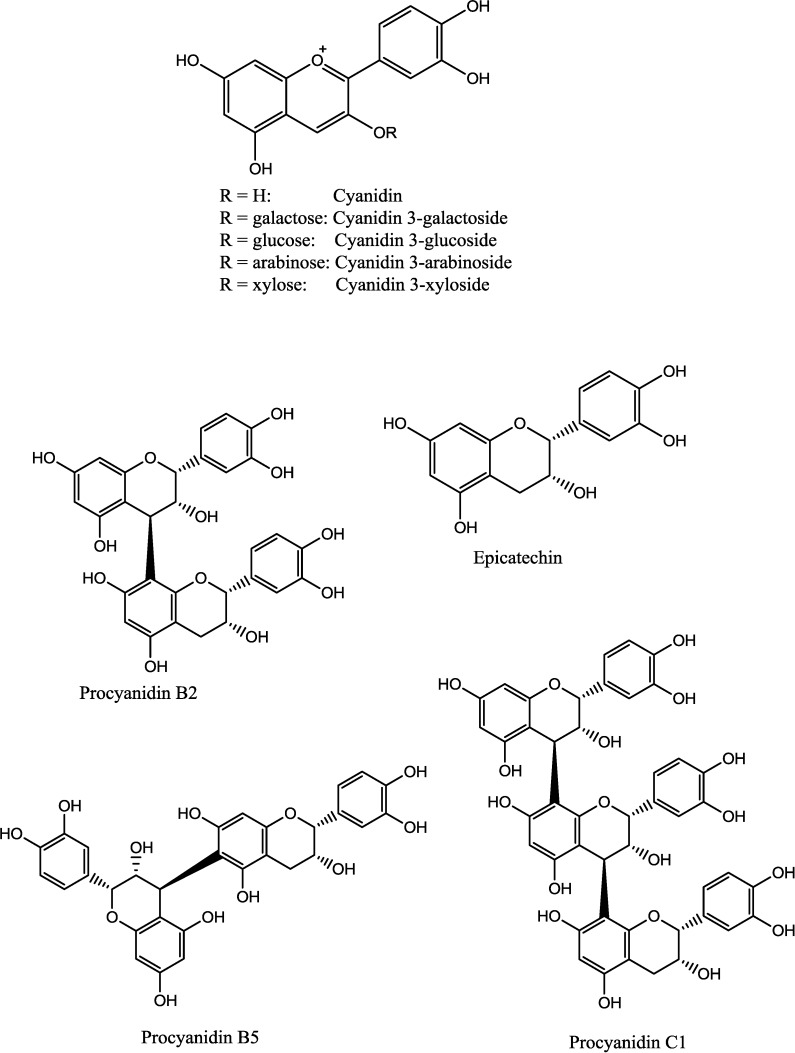
Chemical structures of compounds found in aronia berries.

Aronia berries were extracted with dichloromethane, 96% EtOH and 50% EtOH as previously described [16]. ^1^H-NMR and ^13^C-NMR analyses revealed that proanthocyanidins were present in the 50% EtOH extract, which was therefore further fractionated on a Sephadex LH-20 column to yield subfractions Seph a–g. Thiolysis of the Sephadex fractions revealed an average degree of polymerization (DP) of 7 for Seph d and 34 for Seph g, indicating that the DP of proanthocyanidins found in the subfractions Seph d–g increases with the elution volume. Seph d contained only trace amounts of proanthocyanidins, whereas the majority of proanthocyanidins was found in Seph g. Furthermore, the proanthocyanidins in the subfractions were found to contain epicatechin as the monomeric unit [16,17]. Also, compounds shown in Figure 1, all well known constituents in aronia berries [9,18], were included in this study. Isolated compounds were identified on the basis of their chromatographic and spectroscopic data (TLC, HPLC, LC-MS) and optical rotations [16]. The yield of fractions and content of pure anthocyanins are given in Table 1 and Table 2.

### 2.2. Complement-Fixing Activity

**Figure 2:**
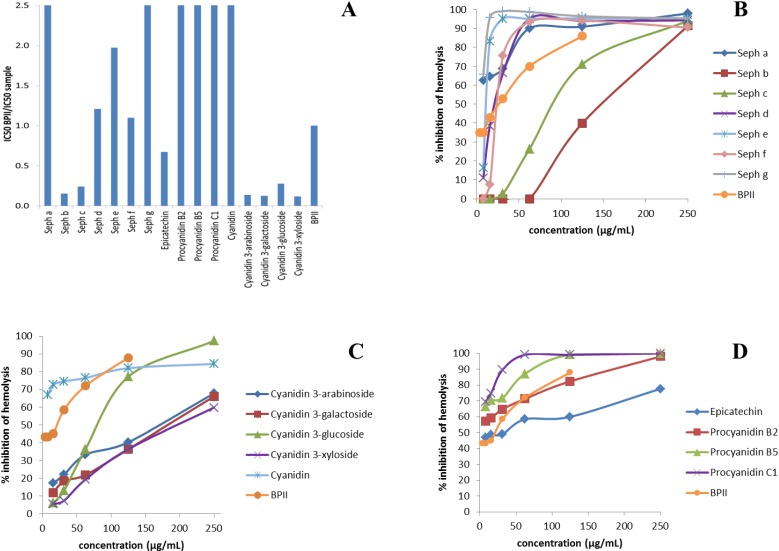
Complement-fixing activity of isolated fractions and phenolic compounds of aronia. (**A**) IC_50_ BPII/IC_50_ ratio for fractions and compounds tested. A high ratio means high complement-fixing activity; (**B**–**D**) Inhibition of hemolysis by subfractions Seph a–g, by anthocyanidins and cyanidin aglycone, and by epicatechin (monomer) and dimeric and trimeric procyanidins. BPII was used as positive control in all these tests. The values represent the mean of duplicate samples (divergence between measurements less than 20%).

The hemolytic complement-fixing test carried out in this study is a commonly used method for screening samples for their influence on the classical complement pathway [3]. The inhibition of hemolysis is a measure of the amount of complement that has reacted with the samples [19]. A pectic polysaccharide from *B. umbraculum* (BPII), used as a positive control, has previously been shown to be highly active in the complement-fixing assay [20,21]. Thus, samples possessing a higher level of activity than BPII are very active as immunomodulators. The anti-complementary effects of isolated fractions and phenolic compounds of aronia were determined *in vitro* (Figure 2). This biological test system can have some day-to-day variation, and thus, the ratio IC_50_ BPII/IC_50_ sample was calculated (Figure 2A). Since the lowest sample concentration used was 7.8 μg/mL and the average IC_50_ value for BPII was 23.1 μg/mL there is an apparent cut-off at 2.5 in Figure 2A. The most active samples may have a greater ratio than this, but all substances which give a ratio of 2.5 or higher can be regarded as highly active. Generally, dimeric and trimeric procyanidins, and polymeric proanthocyanidin-rich fractions were the most active compared to BPII. Since C1 (trimer) and fraction Seph g (polymer) both appear highly active, it does not seem to be a clear correlation between degree of polymerization and complement-fixing effect. The activity of all test samples was concentration-dependent as shown in Figure 2B–D. Among the isolated procyanidins, the trimeric procyanidin C1 possessed higher anti-complementary effect than the dimeric and monomeric flavan-3-ols (Figure 2D), which is in agreement with a previous study by Shahat *et al.* [22]. Interestingly, the difference in complement-fixing activity between procyanidin B2 and the isomeric procyanidin B5, indicates that minor structural differences might influence the ability to modulate the immune system. In addition to the subfractions containing proanthocyanidins (Seph d–g), subfraction Seph a also possessed high complement-fixing activity (Figure 2B). Anthocyanins were much less active than BPII; however, removal of the sugar unit renders these molecules to be fairly efficient as immunomodulators (Figure 2C).

**Table 1:** Yield and composition of fractions from the 50% EtOH extract of aronia berries.

Fraction	Percent of Crude Extract	Main Constituents (from NMR Spectroscopy)
Seph a	33	Carbohydrates
Seph b	11	Carbohydrates
Seph c	1.6	Carbohydrates
Seph d	1.2	Proanthocyanidins
Seph e	1.1	Proanthocyanidins
Seph f	2.5	Proanthocyanidins
Seph g	8.0	Proanthocyanidins

**Table 2:** Content of anthocyanins in aronia berries (as mg cyanidin 3-galactoside equivalents from 100 g fresh weight).

Substance	Content
Cyanidin 3-galactoside	168.4 ± 11.9
Cyanidin 3-glucoside	Trace
Cyanidin 3-arabinoside	83.0 ± 6.6
Cyanidin 3-xyloside	2.7 ± 0.8

### 2.3. Cell Viability and Inhibition of NO Production in LPS-Stimulated RAW 264.7 Macrophage Cells

**Figure 3:**
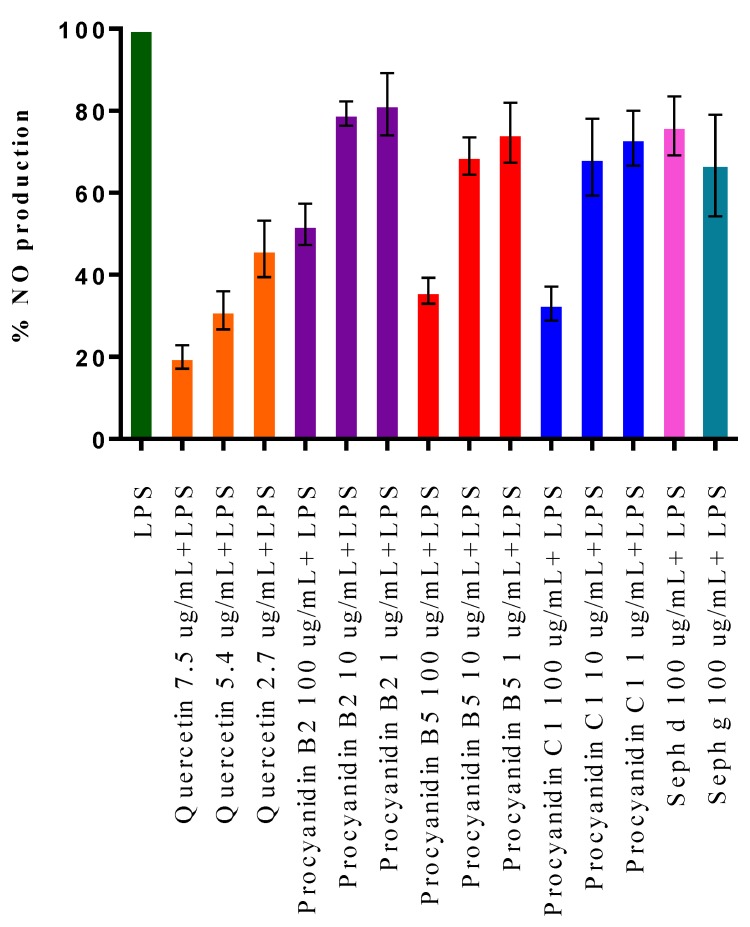
Effects of procyanidins and proanthocyanidin-rich fractions of aronia on NO production in LPS-activated RAW 264.7 macrophages. Quercetin was used as a positive control. Each bar represents the mean ± SD of three individual experiments.

Excessive NO production can result in the development of inflammatory diseases such as rheumatoid arthritis and autoimmune disorders. Thus, inhibition of NO production is a major target for anti-inflammatory agent development [7]. Only fractions and compounds active in the complement-fixing assay were further investigated. Therefore, the effects of subfractions Seph a, d and g and procyanidins B2, B5 and C1 on NO production in murine RAW 264.7 macrophages stimulated with LPS were studied (Figure 3). LPS was used to mimic an inflammatory state by inducing macrophages to produce the inflammatory mediator NO [23]. 

All substances displayed dose-dependent inhibitory effects on LPS-induced NO production, except for Seph a, which did not show any activity even at the highest sample concentration of 100 μg/mL. No significant difference in inhibition of NO production was observed between trimeric procyanidin C1 and dimeric procyanidin B5. In addition, only minor differences were found between procyanidin B5 and isomeric procyanidin B2 (B2: 79% ± 5% of NO production with LPS alone *vs.* 69% ± 8% for B5 at a concentration of 10 μg/mL, not significant; Student’s *t*-test) Subfractions containing polymeric proanthocyanidins, Seph d (DP 7) and Seph g (DP 34), displayed negligible activity below sample concentrations of 100 μg/mL and were thus much less active than the isolated oligomeric procyanidins. Thus, the degree of polymerization for procyanidins and inhibition of NO production in LPS-induced macrophages appears to be negatively correlated, unlike what is the case for the complement-fixing activity.

The effects of isolated procyanidins and subfractions Seph d and Seph g on the viability of RAW 264.7 macrophage cells were determined by a MTS assay, shown in Table 3. Cultured macrophages were treated with the various samples (100 μg/mL) in order to determine if the observed effects of these samples on inhibition of NO production could be caused by cell cytotoxicity. The addition of procyanidin B2, B5 and C1 and subfraction Seph d to macrophage cells did not affect cell proliferation at 100 μg/mL, which confirms that the reduction of NO production was not due to cell death. However, subfraction Seph g decreased the viability of RAW 264.7 cells, while it was also shown to decrease NO production in LPS-activated RAW 264.7 macrophage cells. This reduction in NO production observed for Seph g could therefore be caused by cell cytotoxicity. 

**Table 3:** MTS assay was used to determine cell viability in LPS-stimulated RAW 264.7 treated with procyanidins and proanthocyanidin-rich fractions of aronia. Quercetin was used as a positive control.

Fraction	% Cell Viability
Quercetin 7.5 µg/mL	95
Procyanidin B2 100 µg/mL	117
Procyanidin B5 100 µg/mL	112
Procyanidin C1 100 µg/mL	115
Seph d 100 µg/mL	90
Seph g 100 µg/mL	32

## 3. Experimental Section

### 3.1. Plant Material

*Aronia* berries (*Aronia melanocarpa* (Michx.) Elliott var. Moscow (Rosaceae)) were harvested at Bioforsk Vest Særheim, Klepp, Norway (58°47'N, 5°41'E) in August 2010 and identified by one of the authors (Rune Slimestad). The berries were kept at −20 °C until extraction. A voucher specimen (MB201201) is deposited in the Pharmacognosy section, School of Pharmacy, University of Oslo, Norway. Bark, as a source of procyanidins, was obtained from the same plants as the berries. Branches with a diameter of 1–2 cm were chosen, and the bark was carefully removed. The plant material was cut in pieces and kept at −20 °C until extraction.

### 3.2. Extracts, Fractions and Compounds

Subfractions Seph a–g from aronia berries were available from previous work [16]. Briefly, these fractions were obtained by extraction with 50% EtOH and chromatography over Sephadex LH-20. Pure anthocyanins and proanthocyanidins were likewise available [17]. Cyanidin was prepared as follows: cyanidin 3-galactoside (about 100 mg) was dissolved in MeOH (0.5% HCl, 5 mL) and mixed with 2 M HCl (5 mL). Hydrolysis occurred in a capped glass tube at 100 °C for 30 min. After concentration, cyanidin was isolated from the mixture by elution with 50% MeOH (0.1% HCl) over a bed of Sephadex LH-20 (3 × 40 cm Pyrex column). Purity was determined to be above 97% by HPLC [17]. Epicatechin was purchased from Sigma-Aldrich (St. Louis, MO, USA).

### 3.3. Complement Fixing Assay

The effect of the samples on human complement was measured by method A as described by Michaelsen *et al.* [3]. In this assay, the capacity of the test materials to inhibit complement mediated hemolysis of erythrocytes from sheep, sensitized with rabbit anti-sheep erythrocyte antibodies, was measured. A dilution of human serum resulting in 50% hemolysis of erythrocytes was used as the complement source. Samples were dissolved in 5% MeOH in veronal buffered saline (VBS) pH 7.2 containing 0.2 mM CaCl_2_ and 0.8 mM MgCl_2_ with 2 mg/mL bovine serum albumin (BSA), VBS/BSA. The addition of 5% MeOH to the test samples did not influence hemolysis (results not shown). A Bio-Rad iMark microplate absorbance reader (Hercules, CA, USA) was used for UV-Vis measurements at 405 nm in the complement-fixing assay. Blanks contained VBS/BSA. A dose-response curve was constructed to calculate the concentration of the test sample that gave 50% inhibition (IC_50_) of hemolysis, using PRISM software (GraphPad Software version 5, San Diego, CA, USA). A low IC_50_ value means high complement-fixing capacity. A pectic polysaccharide, BPII, from *Biophytum umbraculum* Welw. (syn. *B. petersianum* Klotzsch) was used as a positive control [20,21]. 

### 3.4. Inhibition of NO Production in LPS-Stimulated RAW 264.7 Macrophage Cells

This assay was performed essentially as in Austarheim *et al.* [24], with some modifications. The mouse macrophage cell line RAW 264.7 was cultured in RPMI 1640 medium supplemented with 10% fetal bovine serum, antibiotics, l-glutamine, and 5 × 10^−5^ M 2-mercaptoethanol. Macrophages at a density of 5 × 10^5^ cells/mL were seeded into 96-well flat-bottomed plates and pre-incubated with the test substance for one hour before the addition of 500 ng/mL LPS (*E. coli* O55:B5, Sigma-Aldrich, St.Louis, MO, USA). Samples were dissolved in 25% MeOH in water. The final concentration of MeOH in the wells did not exceed 0.05% (*v*/*v*). At this MeOH concentration, no significant changes in cytotoxicity and NO production were observed (results not shown). The cells were incubated for 22 h in duplicates with 100, 10 or 1 µg/mL of samples (final concentrations) or medium alone. NO released by activated macrophages is broken down in the medium to nitrite (NO_2_^−^), which can be measured in a colorimetric assay using the Griess reagents (Promega, Madison, WI, USA). The cell free supernatant (50 μL) was mixed with an equal volume of Griess reagent A (1% sulphanilamide in 5% phosphoric acid) and incubated at room temperature in the dark for 10 min. After addition of 50 μL 0.1% *N*-(1-naphthyl) ethylenediamine dihydrochloride in water (Griess reagent B), the absorbance was measured at 540 nm using a Bio-Rad reader (Bio-Rad, Hercules, CA, USA). A serial dilution of NaNO_2_ in medium was used to construct the standard reference curve. Quercetin was used as a positive control. The results are expressed as the average of three individual experiments ± SD.

### 3.5. MTS Assay for Cell Viability

The cytotoxicity of the samples was assessed by a MTS colorimetric assay using CellTiter 96 Aqueous one solution cell proliferation assay kit purchased from Promega (Madison, WI, USA). The assay was carried out according to the manufacturer’s instructions. 

## 4. Conclusions

In this study, we have shown that some polyphenols isolated from *A. melanocarpa* possess activity of potential health benefits as immunomodulators. Cyanidin, procyanidin B2, B5 and C1 and proanthocyanidin-rich fractions were highly active in the complement-fixing assay. In addition, the oligomeric procyanidins displayed dose-dependent inhibitory effects on LPS-induced NO production in murine RAW 264.7 macrophages, without affecting cell proliferation. Our results demonstrate that polyphenolic substances in aronia berries possess immunomodulating and anti-inflammatory activities; however, the clinical relevance of the present *in vitro* observations needs to be further investigated, since activity *in vivo* will depend on the uptake, transport and metabolism of active substances. Higher proanthocyanidins (DP > 3) are not taken up from the GI tract, so the activity of these may be restricted to local effects in the GI system or activity via Peyer’s patches, a well known immunomodulatory mechanism for macromolecules [25].
